# Long-term effects of treatment and response in patients with chronic hepatitis C on quality of life. An international, multicenter, randomized, controlled study

**DOI:** 10.1186/1471-230X-12-11

**Published:** 2012-01-31

**Authors:** Geert Bezemer, Arthur R Van Gool, Elke Verheij-Hart, Bettina E Hansen, Yoav Lurie, Juan I Esteban, Martin Lagging, Francesco Negro, Stefan Zeuzem, Carlo Ferrari, Jean-Michel Pawlotsky, Avidan U Neumann, Solko W Schalm, Robert J de Knegt

**Affiliations:** 1 Dpt. Gastroenterology & Hepatology, Erasmus University Medical Center, Rotterdam, the Netherlands; 2 Yulius Academy, Yulius, Organization for Mental Health, Rotterdam, the Netherlands; 3 Dpt. Gastroenterology, Sourasky Medical Center, Tel-Aviv, Israel; 4 Dpt. Internal Medicine-Hepatology, Hospital General Vall d'Hebron, Barcelona, Spain; 5 Dpt. Infectious Diseases, University of Gothenburg, Gothenborg, Sweden; 6 Dpt. Gastroenterology & Hepatology, Hospital University of Genève, Genève, Switzerland; 7 Dpt. Gastroenterology & Hepatology, Johann Wolfgang Goethe Hospital, Frankfurt, Germany; 8 Dpt. Infectious Diseases and Hepatology, Azienda Ospedaliera di Parma, Parma, Italy; 9 Dpt. Virology, Hopital Henri Mondor - Université Paris XII, Creteil, France; 10 Mina and Everard Goodman Faculty of Life Sciences, Bar-Ilan University, Ramat-Gan, Israel

**Keywords:** health related quality of life, hepatitis C, peginterferon

## Abstract

**Background:**

Hepatitis C decreases health related quality of life (HRQL) which is further diminished by antiviral therapy. HRQL improves after successful treatment. This trial explores the course of and factors associated with HRQL in patients given individualized or standard treatment based on early treatment response (Ditto-study).

**Methods:**

The Short Form (SF)-36 Health Survey was administered at baseline (n = 192) and 24 weeks after the end of therapy (n = 128).

**Results:**

At baseline HRQL was influenced by age, participating center, severity of liver disease and income. Exploring the course of HRQL (scores at follow up minus baseline), only the dimension general health increased. In this dimension patients with a relapse or sustained response differed from non-responders. Men and women differed in the dimension bodily pain. Treatment schedule did not influence the course of HRQL.

**Conclusions:**

Main determinants of HRQL were severity of liver disease, age, gender, participating center and response to treatment. Our results do not exclude a more profound negative impact of individualized treatment compared to standard, possibly caused by higher doses and extended treatment duration in the individualized group. Antiviral therapy might have a more intense and more prolonged negative impact on females.

## Background

Patients chronically infected with hepatitis C virus (HCV) have a decreased health related quality of life (HRQL) compared to the general population [1,2]. The impact of the disorder is comparable with other stressful life events and chronic diseases, like diabetes [3]. In part, the reduction in HRQL is due to the mental components of HRQL. With regard to these mental components, patients aware of their diagnosis have a more reduced HRQL than those who are unaware [4]. Furthermore, many HCV patients have a previous or ongoing addiction and/or psychiatric problems, reflected in lower HRQL. In addition, patients with hepatitis C are stigmatized in society and the majority of the population of hepatitis C patients has a lower social economic status compared to the general population [5].

The reduction of HRQL is probably also due to physical and psychiatric symptoms as a direct consequence of this chronic infection and its sequelae (such as cirrhosis). The chronic inflammation is believed to signal the brain and to give rise to neurovegetative symptoms (e.g. malaise and fatigue) and to amongst others depression and concentration difficulties [6]. Possibly, also the brain itself is infected by HCV [7].

Finally, treatment of chronic HCV with (peg)interferon-alpha ((PEG)IFN) and ribavirin (RBV) further diminishes HRQL due to its side-effects. The introduction of PEGIFN provided a significant improvement over standard IFN, with the result that the decrease of HRQL during treatment with PEGIFN is less than with standard IFN [8-11]. In case of successful treatment (obtaining a sustained virological response (SVR)) an improvement of HRQL-scores is observed [2,8-10].

**Figure 1:**
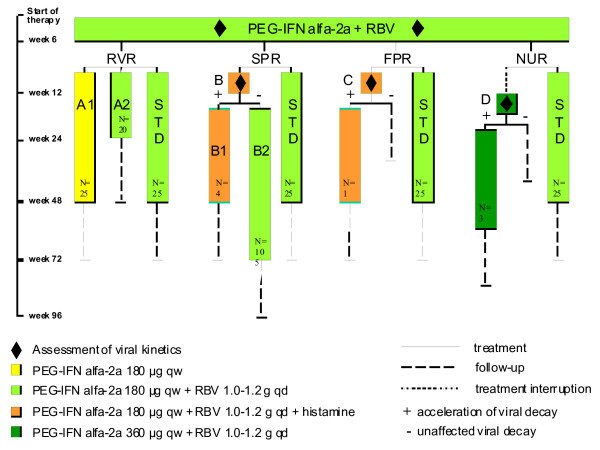
**Treatment allocation of patients with chronic hepatitis C according to initial virologic response pattern in the Ditto study**. Abbreviations: Ribavirin (RBV), Rapid viral response (RVR), Slow partial response (SPR), Flat partial response (FPR), null response (NUR), Standard combination therapy (STD).

In the Ditto-study [12] a dynamically individualized treatment schedule depending on the on-treatment response was compared to a standard combination therapy with PEGIFN alfa-2a (180 μg qw) plus RBV (1000-1200 mg qd) for 48 weeks (Figure 1). The primary aim of the Ditto study was to improve the SVR rate by individualizing the treatment schedule, but this could not be established.

This large scale, multi-centre, international trial, firstly, provides the opportunity to explore a variety of factors associated with HRQL in HCV patients at baseline (treatment naïve; t = 0). Secondly, although type of treatment, treatment intensity and treatment duration were heterogeneous this study enabled us to investigate the course of HRQL during PEGIFN-based treatment and to add to the existing knowledge on the impact of cytokine-based antiviral treatment on HRQL. (E.g. do patients recover 24 weeks after completion of therapy (follow up) and which factors are associated with the course of HRQL?)

## Methods

### Patients

273 patients were included in the study, after informed consent was obtained, and 270 patients were randomized after 6 weeks of standard treatment. The ethical review boards of the participating centers approved the study. After randomization, 134 patients received standard treatment and 136 patients were treated according to an individualized treatment schedule depending on the response at week 4 and 12 of treatment. 216 patients completed the treatment per protocol and 249 patients completed the end of follow up. At t = 0 HRQL was measured in 192 patients (not every center participated in the HRQL-analysis) and at follow up (24 weeks after completion of therapy) in 128 patients. In 120 patients, HRQL could be assessed at both time points.

### Study Design

This phase III, open-label, randomized, multicenter trial was conducted by the DITTO-HCV study group between February 2001 and November 2003 at 9 centers in France, Germany, Greece, Israel, Italy, the Netherlands, Spain, Sweden, and Switzerland.

### Assessments

The Short Form (SF)-36 Health Survey was used to measure HRQL. The SF-36 generates a profile of HRQL outcomes by measuring health across eight different dimensions: physical functioning, role limitation because of physical health, social functioning, vitality, bodily pain, mental health, role limitation because of emotional problems and general health. Responses to each question within a dimension are combined to generate a score from 0 to 100, where 100 indicates "good health". The eight multi-item subscales were also converted into a physical component summary scale and a mental component summary scale [13]. Demographic (such as age, gender, and income in Euros) and disease related factors were assessed before start of treatment. At the moment the SF-36 was completed at follow up both patients and the treating physician were unaware of the response to the treatment because HCV-RNA testing results were not known yet, however non-responders were aware of their (positive) HCV-RNA status at relevant time points during treatment.

### Data analysis

#### Power calculation

The HRQL-analysis was part of the main study which was powered on significant differences in SVR-rates between standard and individualized treatment. Therefore no separate power analysis was performed for this analysis.

#### Determinants of HRQL before treatment

To investigate the influence of different characteristics such as age, grade of fibrosis, and income in Euros, t-test analysis for dichotomous categorical, univariate analysis for multicategorical and correlation analysis (Spearman) for continuous data were performed (n = 192).

#### Course of HRQL

The differences between HRQL at t = 0 and follow up were investigated with analysis of variance (ANOVA) and t-tests for Equality of Means (n = 120).

#### Explanatory variables on the course of HRQL

##### a) Effects of response to treatment on the course of HRQL

The course of HRQL was analyzed separately for patients with SVR, relapse or non-response with t-tests. Comparison between patients with SVR, with relapse and non-response was assessed by means of ANOVA.

##### b) Effects of treatment schedule

The mean differences in quality of life between t = 0 and follow up were compared between patients treated with standard and individualized treatment using ANOVA and t-tests for Equality of Means. Also the mean differences were compared between the groups with RVR (A1, A2 and standard treatment with RVR) and the groups B2, D and A2 (extended versus shortened therapy, see Figure 1).

##### c) Effects of patient characteristics on the course of HRQL

With t-test analysis for dichotomous categorical, univariate analysis for multicategorical and correlation analysis (Spearman) for continuous data, the influence on the differences between HRQL at t = 0 and follow up was determined for the factors age (< 20, 20-29, 30-39, 40-49, 50-59, 60-69 years), gender, participating center, presence of cirrhosis, genotype (1, 4, and 5 versus 2 and 3) and income in Euros.

##### d) Multivariate analysis

For further exploration of the influence of the different factors mentioned above on the course of HRQL, a multivariate analysis was performed with a correction for age and baseline values of the SF-36.

## Results

Baseline host and virus-related variables were similar in the standard and the individualized treatment groups (data not shown).

### Determinants of HRQL before treatment

**Figure 2:**
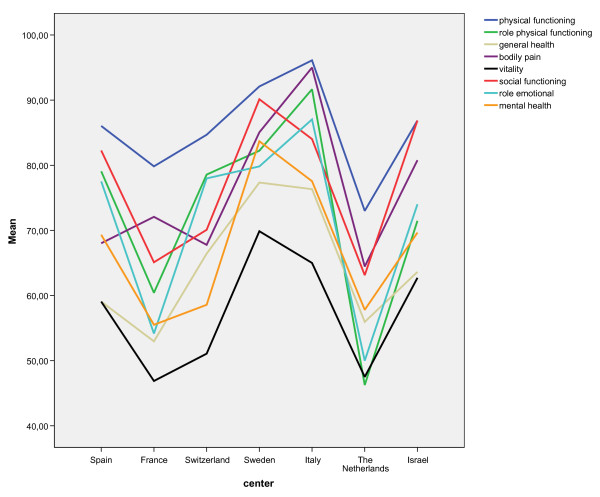
**Mean scores of the different SF36 dimensions at t = 0 from the participating centers**. Barcelona; Spain, Paris; France, Geneva; Switzerland, Gothenburg; Sweden, Parma; Italy, Rotterdam; The Netherlands, Rehovot; Israel.

HRQL varied with age, participating center, severity of liver disease and income in Euros (Table 1). As might be expected, younger patients had a better performance compared to older patients, especially on the physical components of HRQL. Surprisingly, cirrhotic patients had a better performance than non-cirrhotic patients, having significantly higher scores on several dimensions of the SF-36, mostly on mental components. Higher income in Euros had a weak positive effect on the physical component summary scale and role limitation because of physical health scale of HRQL, with a correlation coefficient of 0.30 (p = .003) and 0.23 (p = .002) respectively. Finally, HRQL differed among the different participating centers: the mental component summary scale and the dimensions physical functioning, role limitation because of physical health, general health, bodily pain, vitality, social functioning and mental health were all significantly different between the participating centers (Figure 2).

**Table 1:** Determinants of Health Related Quality of Life on t = 0 and course of Health Related Quality of Life

	Subscale	t = 0		P-value	Course (follow up - t = 0)		P-value
**Age**		Age category (20-29, 30-39, 40-9, 50-59)			Age category (20-29, 30-39, 40-49,50-59)		

	Physical functioning	90.6, 93.1, 86.1, 78.1		0.01			

	**Subscale**	**t = 0**		**P-value**	**Course** **(follow up - t = 0)**		**P-value**

**Cirrhosis **No/Yes		No	Yes		No	Yes	

	Mental component summary scale	44.7	53.7	0.001			

	General health	61.3	73.5	0.003			

	Bodily pain	74.2	88.4	0.04			

	Role limitation because of emotional problems	68.9	88.1	0.05			

	Mental health	66.4	80.4	0.0004			

	**Subscale**	**t = 0**		**P-value**	**Course** **(follow up - t = 0)**		**P-value**

**Income in Euros**	Subscale SF-36	Correlation coefficient		P-value	Correlation coefficient		P-value

	Physical component summary scale	0.30		0.003			

	Role limitation because of physical health	0.23		0.002			

	Vitality				.30		0.02

	Bodily pain	0.18		0.07			

	Mental health				.21		0.03

	**Subscale**	**t = 0**		**P-value**	**Course** **(Follow up - t = 0)**		**P-value**

**Gender **(M/F)		M	F		M	F	

	Bodily pain				+ 4.1	- 7.2	0.02

	Social functioning				+ 6.3	- 5.8	0.008

	Vitality				+ 7.2	- 2.7	0.03

	Role limitation because of emotional problems				+ 9.3	- 8.3	0.05

Results of statistical analysis of the influence of age (ANOVA), presence of cirrhosis (t-test), gender (t-test) and income in Euros (correlation analysis: Spearman) on Health Related Quality of Life at start of treatment (t = 0) and the course of Health Related Quality of Life (scores at 24 weeks after treatment (Follow up) minus scores at baseline). Data were shown if relationships were statistically significant.

### Course of HRQL

General health improved significantly at follow up compared to general health at start of treatment (mean improvement 3.5, p = .04). The subscale physical functioning tended to worsen with a mean decrease of - 3.4 (p = .06) compared to t = 0. Other dimensions did not change.

### Explanatory variables on the differences between HRQL at baseline and at follow up

#### a) Effect of response to treatment on course of HRQL

In patients with SVR, as well as with a relapse or non-response, HRQL did not change compared to t = 0 after treatment with PEGIFN antiviral therapy, except for an increase in social functioning in sustained viral responders (78.8 at baseline, 83.3 at follow up, p = .04). Among the different groups changes compared to baseline in the dimensions general health differed significantly (p = .02) between patients with a relapse (+ 5.3), SVR (+ 6.1) and non-response (- 6.3) to treatment.

#### b) Effect of treatment schedule on course of HRQL

No significant differences were observed in changes in the different dimensions of the SF-36 between standard and individualized treatment. A sub-analysis comparing responders and non-responders separately in standard and individualized treatment showed no difference, either. Patients treated for 24 weeks and patients treated with double dose PEGIFN showed a decline on the dimensions mental health whereas patients treated for 72 weeks had an increase on this dimension: respectively - 7.9, - 2.0 and + 18.5.

#### c) Effects of patient characteristics on course of HRQL

**Figure 3:**
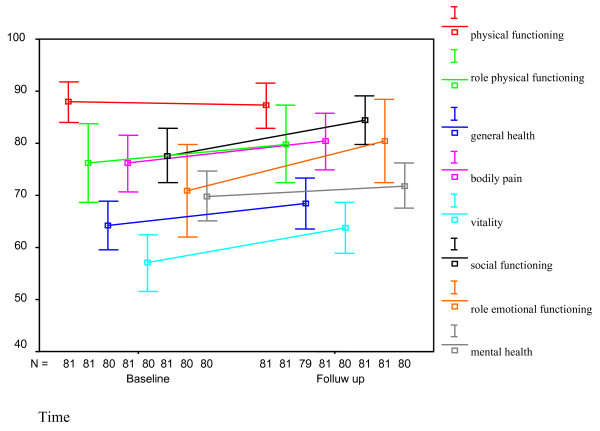
**Mean scores with 95% confidence intervals of the different dimensions of the SF-36 in men at baseline and at 24 weeks after completion of treatment (follow up)**.

**Figure 4:**
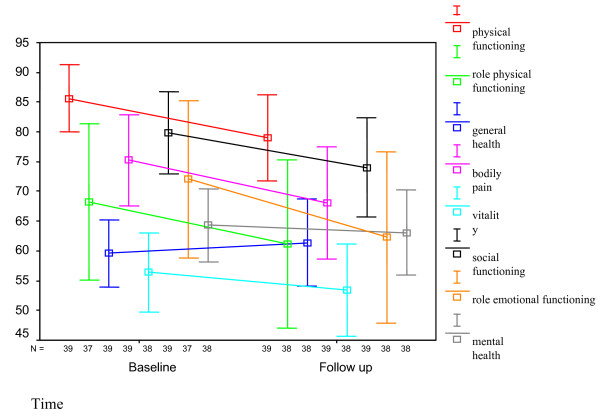
**Mean scores with 95% confidence intervals of the different dimensions of the SF-36 in females at baseline and at 24 weeks after completion of treatment (follow up)**.

Neither age, genotype nor grade of fibrosis had an influence on changes in the different dimensions of HRQL. A significant difference was seen between men and women in the dimensions bodily pain (males: + 4.1, females: - 7.2, p = .02), social functioning (males: + 6.3, females: - 5.8, p = .008), vitality (males: + 7.2, females: - 2.7, p = .03) and role limitations due to emotional problems (males: + 9.3, females: - 8.3, p = .05). There was a weak significant positive correlation between income in Euros and increase in vitality and mental health between baseline and follow up: correlation coefficient .30 (p = .02) for vitality and .21 (p = .03) for mental health. See Figure 3 and 4.

#### d) Multivariate analysis

Also in a multivariate analysis gender was a significant factor on the course of HRQL. All different dimensions, except for physical component summary scale and general health, were significantly different between men and women (Table 2). Response to treatment was again significantly influencing general health.

**Table 2:** Multivariate analysis

	Physical component summary scale	Mental component summary scale	Physical functioning	Role limitation because of physical health	General health	Bodily pain	Vitality	Social functioning	Role limitation because of emotional problems	Mental health
Gender		.043	.050	.030		.006	.009	.001	.024	.023
SVR					.047					
Standard vs Individualized treatment	.031					.019	.027	.031		.075

Multivariate analysis (corrected for baseline value and age) of the influence of gender, response to treatment and kind of therapy on the mean difference between baseline and follow up (course of Health Related Quality of Life) of the different dimensions of the SF-36.

Only significant p-values are shown in this table.

Abbreviations: Sustained Virological Response (SVR).

In the multivariate analysis, individualized treatment had a significantly negative influence on the course of HRQL on the dimensions physical component summary scale, bodily pain, vitality and social functioning, compared to patients treated with standard treatment (Table 2).

The presence or absence of cirrhosis and grade of fibrosis did not play a role in the course of HRQL in this analysis. Also different kind of genotypes (1, 4 and 5 versus 2 and 3) did not influence changes in HRQL.

## Discussion

In this study we explored the influence of PEGIFN-based antiviral treatment with various regimens and of patients characteristics on HRQL before as well as at 24 weeks after completion of treatment. Main determinants of HRQL in this study were the severity of liver disease (non-cirrhosis vs. cirrhosis), age, gender and participating center and - to some extent - response to treatment. With regard to the severity of the liver disease, patients with cirrhosis did better compared to those without cirrhosis. We are unable to explain this difference.

In our study almost all dimensions of the SF-36 did not change regardless of response, indicative of a satisfying recovery of patients after cytokine-based therapy. However, a significant increase was found on the subscale general health despite a therapy-induced worsening of the subscale physical functioning. This improvement was seen in patients with both SVR and relapse, whereas non-responders showed a decrease in general health. In our study, interestingly the relapsers who were ignorant of treatment effect when filling in the questionnaire, had a far more higher score on general health compared to the non-responders who were aware of their previous non-response to therapy. This underlines the mental aspects of the impact on HRQL in this disease [14]. The observed recovery in HRQL is in line with previous studies reporting an improvement on (almost) all different dimensions of the SF-36 after a successful treatment with PEGIFN and RBV compared to baseline scores [2,8-10].

The presence of cirrhosis did not influence the course of HRQL during treatment, but patients with a cirrhosis had higher scores on some, mostly mental, dimensions of the SF-36 at t = 0 and at follow up, which is in contrast with earlier studies [8,15]. Patients with decompensated cirrhosis were excluded to participate in the study, but still we cannot explain this difference. Higher age was associated with a decrease in HRQL, according to literature data [16].

Men and women differed in the course of HRQL with an increase on scores for men on several dimensions and women experiencing a decrease. These differences between men and women were also significant in a multivariate analysis for all dimensions except for general health. In the general population, men report a higher HRQL [16-18], a finding possibly of relevance for our findings. But also, therapy with PEGIFN and RBV might have a more intense and more prolonged impact on females. Studies show more severe anemia in women during treatment with PEGIFN and RBV [19]. As anemia plays an important role in HRQL during antiviral treatment this could be one of the explanations for the observed difference [20]. With the fixed doses of PEGIFN women may be treated with higher doses of PEGIFN per kg, which may contribute to more side-effects and a lower HRQL during treatment and at 24-weeks follow-up. To our knowledge, only one study found sex differences in recovery, with males doing slightly worse [21].

HRQL at baseline was found to be differing between the participating centers (in this case different countries/cultural regions). In the study by Ware et al [9] a similar observation was made. Although much attention has been devoted to assure the comparability between different cultures and languages, this observation suggests that studies from different countries concerning side effects (for instance decrease of HRQL, but also psychopathology) should be interpreted with caution [22,23].

An important limitation of this study is the large number of variables in relation to the number of participants and the many different treatment schedules. Also the relative high number of drop-outs with data of 192 patients at baseline and of 128 patients at follow up could be of importance, however non-responders or relapsers were not significantly more non-compliant compared to responders (data not shown). In retrospect an additional time point at the end of treatment would have given more insight.

## Conclusion

In conclusion: our findings support the observation, that in general patients exposed to PEGIFN-based treatment do recover to their pre-treatment level of baseline, six months after completion of antiviral therapy or even surpass that level. This underlines again the safe profile of this intensive treatment, irrespective of the used dosage and/or duration in this study. Before treatment and after treatment with PEGIFN in patients chronically infected with HCV health related quality of life is mostly influenced by presence of cirrhosis, age, gender, participating center (or country) and response to treatment. Also, awareness of response status to therapy seems important.

## Competing interests

This study was supported by the European Community (QLK2-2000-00836), Hoffmann La-Roche and Maxim Pharmaceuticals.

In addition and in relation to this study, the authors would like to disclose the following:

Geert Bezemer, nothing to disclose

Arthur R. Van Gool, nothing to disclose

Elke Verheij-Hart, nothing to disclose

Bettina E. Hansen, nothing to disclose

Yoav Lurie, nothing to disclose

Juan I. Esteban, nothing to disclose

Martin Lagging, nothing to disclose

Francesco Negro, nothing to disclose

Stefan Zeuzem, nothing to disclose

Carlo Ferrari, nothing to disclose

Jean-Michel Pwalotsky, nothing to disclose

Avidan Neumann, nothing to disclose

Solko W. Schalm, nothing to disclose

Robert J. de Knegt, nothing to disclose

## Authors' contributions

GB contributed to the design of the study, acquired and analysed the data, and drafted the manuscript; AG contributed to the design of the study, analysis of the data and was involved in drafting the masnuscript; EVH contributed to the analysis of the data; BH contributed to the statistical analysis of the data; YL contributed to data-acquirement and revision of the manuscript; JE contributed to data-acquirement and revision of the manuscript; ML contributed to data-acquirement and revision of the manuscript; FN contributed to data-acquirement and revision of the manuscript; SZ contributed to design of the study, data-acquirement and revision of the manuscript, CF contributed to data-acquirement and revision of the manuscript; JMP contributed to data-acquirement and revision of the manuscript; AUN contributed to data acquirement and revision of the manuscript; SWS contributed to the design of the study, data-acquirement and revision of the manuscript; RJK contributed to the design of the study, data-requirement and analysis, and drafting the manuscript. All authors have read and approved the final manuscript.

## Pre-publication history

The pre-publication history for this paper can be accessed here:

http://www.biomedcentral.com/1471-230X/12/11/prepub
